# Homolytic Anemia of the Pernicious Type with Visceral Hemosiderosis in Tuberculous Subjects

**Published:** 1913-09

**Authors:** 


Homolytic Anemia of the Pernicious Type With Visceral Hemosiderosis in Tuberculous Subjects. By G. Roque, J. Chalier and L. Nove Josserand. (Arch. des Mal. du Coeur des Vaisseaux, et du Sang, 1913, VI, 23. The authors report the case of a tuberculous patient, aged 35 years, who, upon hemotalogic examination showed an unusual blood picture: Erythrocytes, 1,147,000; leukocytes, 2945; hemoglobin, 15% (sahli); Color index, 0.65. Differential Count: Polymorphonuclear neutrophiles, 59.0%; lymphocytes, 24.0%; large mononuclears, 7.0%; transitionals, 3.0%; eosinophiles, 2.0%; myelocytes, 5.0%.

The Arneth formula was strongly deflected to the left. The resistance of the red cells was 0.46. There was marked anisocytosis, poikilocytosis, and slight polychromatophilia and granular degeneration. One or two negloblasts were seen to each 100 leukocytes. Coagulation was immediate. The serum was strongly hemolytic. At the autopsy the liver, the spleen and the kidneys were found to contain iron in the following proportions: Liver, 0.269 gm. per 1000; spleen, 0.367 gm. per 1000; kidneys, 0.67 gm. per 1000. The authors consider the anemia to be due to hemolysis rather than to a want of hematopoiesis. The serum of the patient contained an autolysin as well as an isolysin.

